# Editorial: Cannabis and emotion processing

**DOI:** 10.3389/fpsyg.2022.1039982

**Published:** 2022-10-17

**Authors:** Lucy J. Troup, Anita Cservenka

**Affiliations:** ^1^Division of Psychology, School of Education and Social Science, University of the West of Scotland, Paisley, United Kingdom; ^2^School of Psychological Science, Oregon State University, Corvallis, OR, United States

**Keywords:** cannabis, marijuana, emotion, emotion recognition, emotion regulation, stress

Worldwide attitudes and legislation toward cannabis have changed over the past several decades. For example, there has been a significant shift in legislation controlling cannabis as a recreational drug and as medicine both within the United States and the United Kingdom. Many countries now have laws in place for medical cannabis prescribing, and some for recreational use. Despite these changes and the lowered perception of risk related to cannabis use (Salloum et al., 2018), there are still significant gaps in our understanding of the effects of cannabis both physiologically and psychologically. In particular, there is a growing interest in understanding the relationship between cannabis use and emotion processing (Mackenzie and Cservenka, 2021) as individuals using cannabis often have co-occurring negative affect and mood symptoms (Troup et al., 2016a), and show differences in emotion recognition and brain activity in response to emotional stimuli, compared to healthy controls (Platt et al., 2010; Hindocha et al., 2014; Bayrakci et al., 2015; Troup et al., 2016b). These studies suggest that impairments in emotion processing both at the behavioral and neural levels could be related to the maintenance of chronic cannabis use. Thus, this collection of published research aims to bring together new findings related to cannabis use and emotion processing in order to increase our understanding of potential targets for prevention, intervention, and treatment studies.

Sullivan et al. conducted a functional neuroimaging study using a Go/No-go task, in which they report that abstinent young adult cannabis users showed less rostral anterior cingulate (rACC) activity compared to healthy controls during fearful response inhibition. These findings suggest potentially aberrant emotion regulation and/or emotion processing brain activity in cannabis users, consistent with other researchers' findings (Troup et al., 2016b; Zimmermann et al., 2017; Torrence et al., 2019). Female, relative to male, abstinent cannabis users also exhibited reduced connectivity of rACC with the cerebellum. This highlights potential sex differences in the effects of cannabis on emotion processing that could suggest greater female vulnerability to the negative effects of cannabis use.

Blair et al. examine the role of cannabis use disorder severity on emotion recognition in at-risk youth with conduct disorder (CD) problems, as youth with CD have previously shown reduced emotion expression recognition abilities (Fairchild et al., 2009). The authors find that both CD and cannabis use disorder severity are independently negatively associated with emotion recognition accuracy, suggesting that cannabis use may increase the risk for anti-social and aggressive behavior.

Since healthy socio-emotional processing may be important to substance use disorder (SUD) recovery (Anand et al., 2017), Bjork et al. investigate the role of different SUDs, including cannabis use disorder (CaUD), on social information processing with a Go/No-go task. Participants with CaUD had reduced hit rates to emotionless faces compared to healthy controls, and greater false alarm rates in response to non-target emotional faces (particularly happy faces), suggesting a potential bias toward positively valenced emotional faces. The authors suggest that this approach behavior to positive social stimuli could be used as an important non-drug reward for recovery from CaUD. These findings also support previous research suggesting altered processing of happy faces in cannabis users (Torrence et al., 2019).

In other contributions to this special topic, al'Absi and Allen review the literature on the relationship between acute and chronic cannabis effects on the regulation of stress responses. In particular, they report sex differences as a variable of interest considering significant increases in women using cannabis during pregnancy in response to stress. Additionally, they highlight the role of emotion processing disorders and their comorbidity with cannabis use to emphasize the importance of studying the role of stress in cannabis use within specific subpopulations.

Similarly, Cavalli and Cservenka report on the role of stress in problematic cannabis use. Their data show that more stressful life events and less perceived stress are associated with more problematic cannabis use at greater levels of emotion dysregulation. These findings indicate that emotion dysregulation is an important moderator of the relationship between different measurements of stress and problematic cannabis use.

The studies in this Research Topic suggest that cannabis use may be related to emotion processing and stress regulation impairments at both a behavioral and neural level. Despite evidence that cannabis is used in the context of a potential treatment for emotion processing disorders (Erridge et al., 2021), it seems clear from the studies in this Research Topic that cannabis use may be related to atypical emotion processing and regulation. One of the important factors that has been highlighted in the research reported here is understanding individual differences. For example, future studies should continue to investigate the role of psychiatric co-morbidities, gender, and SUD status, as possible variables that may influence the association between cannabis and emotion processing. Furthermore, types of stress and the valence of emotional stimuli may be important variables to consider in future research. In other words, we must look at cannabis use in the context of emotion processing in all of its many facets as well as highlight the role of exogenous variables and how they affect emotion processing to better develop targets for prevention, intervention, and treatment studies.

## Author contributions

All authors listed have made a substantial, direct, and intellectual contribution to the work and approved it for publication.

## Conflict of interest

The authors declare that the research was conducted in the absence of any commercial or financial relationships that could be construed as a potential conflict of interest.

## Publisher's note

All claims expressed in this article are solely those of the authors and do not necessarily represent those of their affiliated organizations, or those of the publisher, the editors and the reviewers. Any product that may be evaluated in this article, or claim that may be made by its manufacturer, is not guaranteed or endorsed by the publisher.
